# Combined proximal humerus fracture and acromioclavicular joint injury: A case report

**DOI:** 10.1016/j.ijscr.2020.02.038

**Published:** 2020-02-22

**Authors:** Chaiwat Chuaychoosakoon, Prapakorn Klabklay

**Affiliations:** Department of Orthopaedic Surgery and Physical Medicine, Faculty of Medicine, Prince of Songkla University, 15 Karnjanavanich Road, Hat Yai, Songkhla, 90112, Thailand

**Keywords:** Proximal humerus fracture, Acromioclavicular joint injury, Combined, Supine position

## Abstract

**Introduction:**

Both the proximal humerus fracture and the acromioclavicular (AC) joint injury are commonly found in shoulder girdle injuries but there are no reports of them presenting together. The mechanism of the AC joint injury is similar to that of the proximal humerus fracture, a lateral impact to the shoulder girdle, but the arm positions are different, as the AC injury usually involves an adducted arm while the proximal humerus fracture normally occurs with the arm in a neutral position. Herein we report, to our knowledge, the first case of a combined proximal humerus fracture and AC joint injury.

**Case presentation:**

A 40-year-old Thai male presented with right shoulder pain after a motorcycling accident. He was diagnosed as a proximal humerus fracture (Neer 4-part fracture). Open reduction and internal fixation with a Philos plate (Synthes®) in the supine position were then performed. However, an AC joint injury (Rockwood type III) was then noticed on the post-operative X-ray taken in the upright position. We had missed the AC joint injury because all pre-operative imaging had been done only in the supine position.

**Conclusion:**

The combination of these two fracture types is uncommon and has not been previously reported. Our report suggests that in cases of a proximal humerus fracture resulting from a high-energy mechanism, the surgeon should obtain an X-ray or perform fluoroscopy with the patient in the upright or semi-upright position before surgery to avoid missing an AC joint injury.

## Highlights

• The incidence of combined proximal humerus fracture and acromioclavicular joint injury is rare.
• High energy trauma can injure more than one structure.
• In shoulder joint injury, the surgeon can avoid missing a related injury by investigating the patient in the upright position.

## 1 Introduction

Both the proximal humerus fracture and the acromioclavicular (AC) joint injury are common shoulder girdle injuries following high-energy trauma. The incidences of proximal humerus fractures and AC joint injuries are 6% and 9%, respectively [1,2]. The mechanisms of proximal humerus fractures are different between younger and older adults. In the younger adult group, the fracture commonly occurs from a high-energy impact directly on the shoulder, while the older age group usually acquires this type of injury from low-energy loading in an out-stretched hand position. In AC joint injuries, which can affect any age, the mechanism is similar to the younger adults who suffer a proximal humerus fracture from a lateral impact on the shoulder girdle, but while the proximal humerus fracture occurs with the arm in a neutral position, the arm position of an AC joint injury usually results from an impact with the arm in the adducted position [3]. From this reason, a combined proximal humerus fracture with an AC joint injury is unusual. There are no reports in the recent literature of such a combination, and herein we offer the first case report of a combined proximal humerus fracture and acromioclavicular joint injury. This case report was approved by the Research Ethics Committee of Prince of Songkla University (REC 62-411-11-1) and was made according to the SCARE criteria [4].

## 2 Presentation of case

**Fig. 1:**
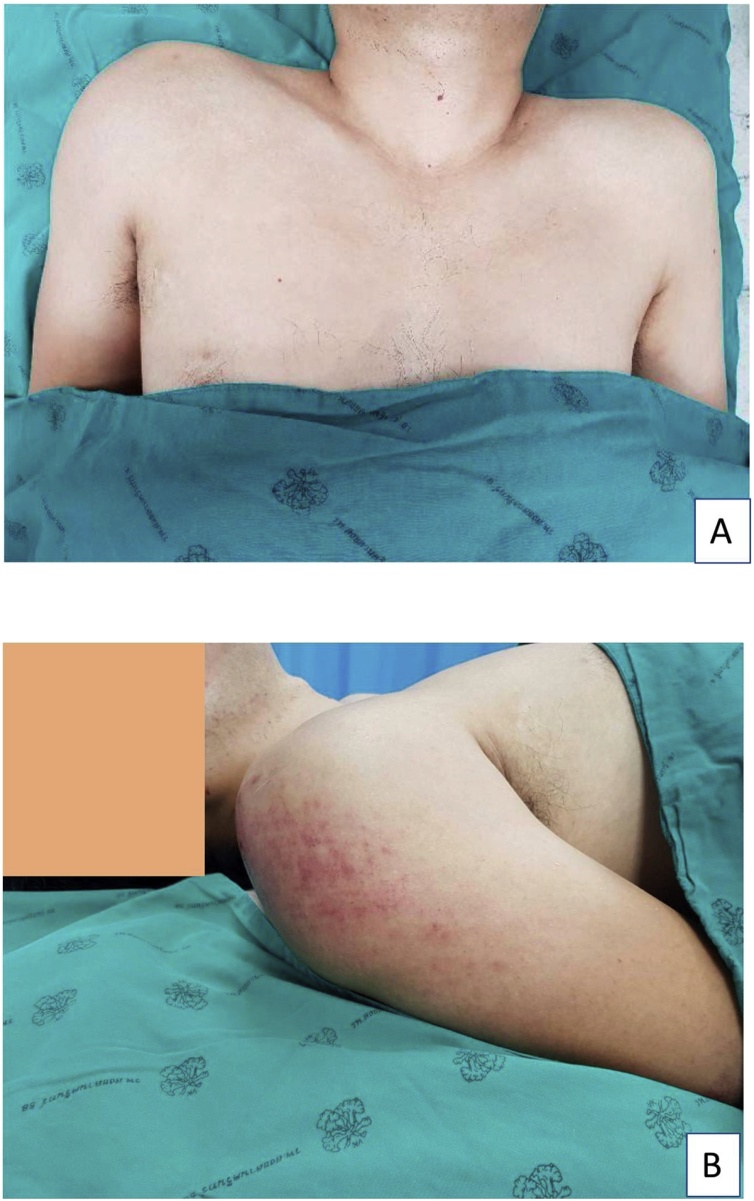
The patient’s initial appearance in (A) anterior view and (B) lateral view.

**Fig. 2:**
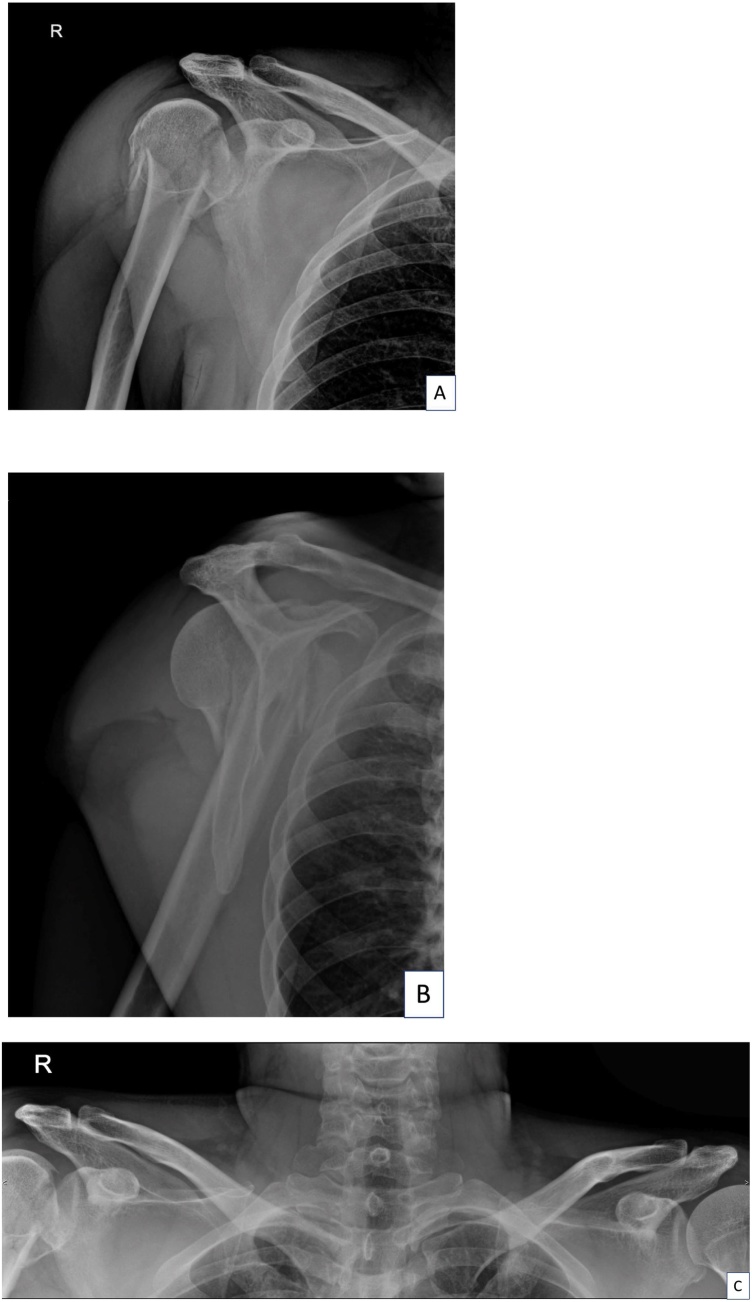
The initial radiographic imaging: (A) anteroposterior view of right shoulder, (B) transcapular view of right shoulder and (C) anteroposterior view of both clavicles.

**Fig. 3:**
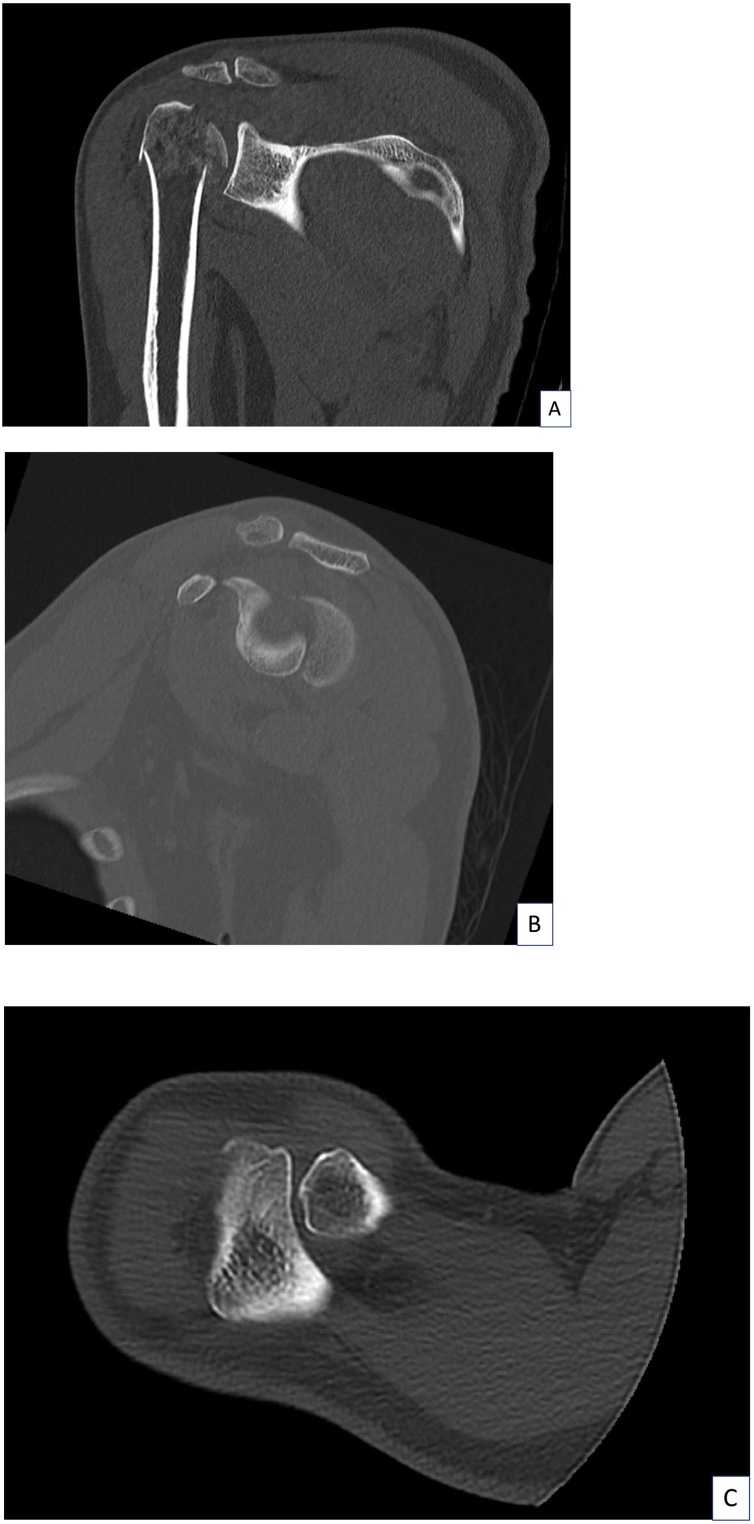
Initial computed tomography: (A) coronal oblique view, (B) sagittal view, and (C) axial view.

**Fig. 4:**
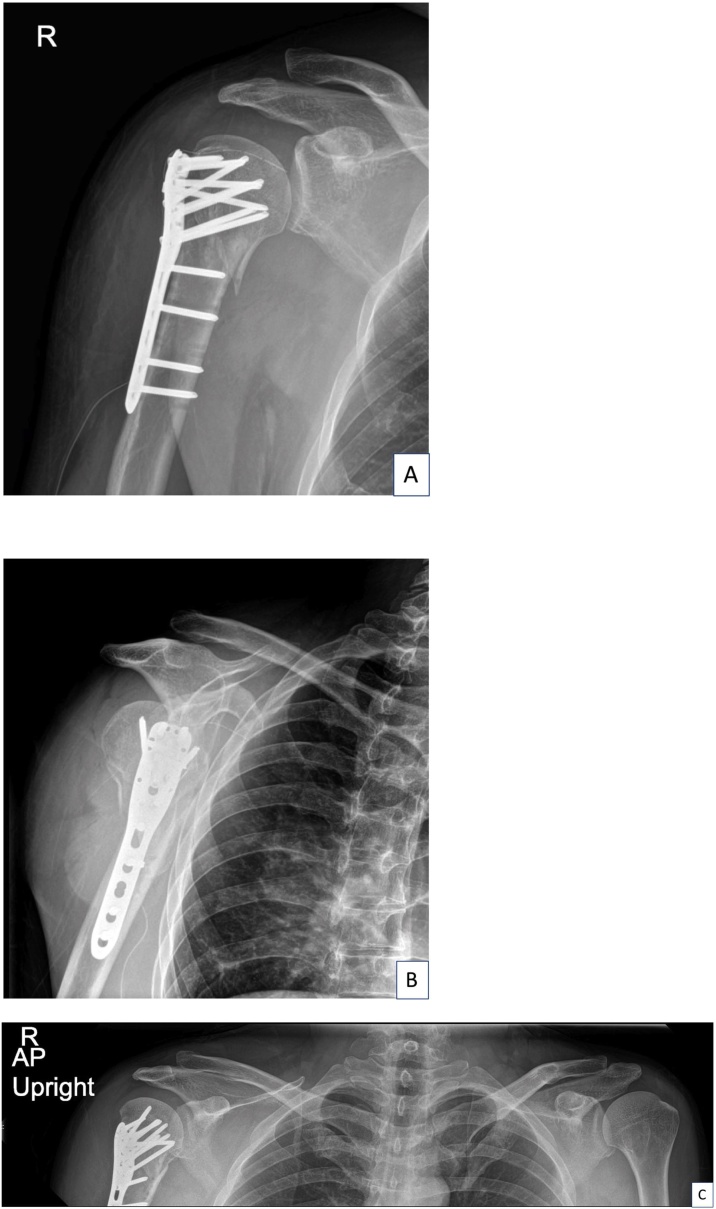
Postoperative radiographic imaging: (A) anteroposterior view of right shoulder, (B) transcapular view of right shoulder, and (C) anteroposterior view of both clavicles.

**Fig. 5:**
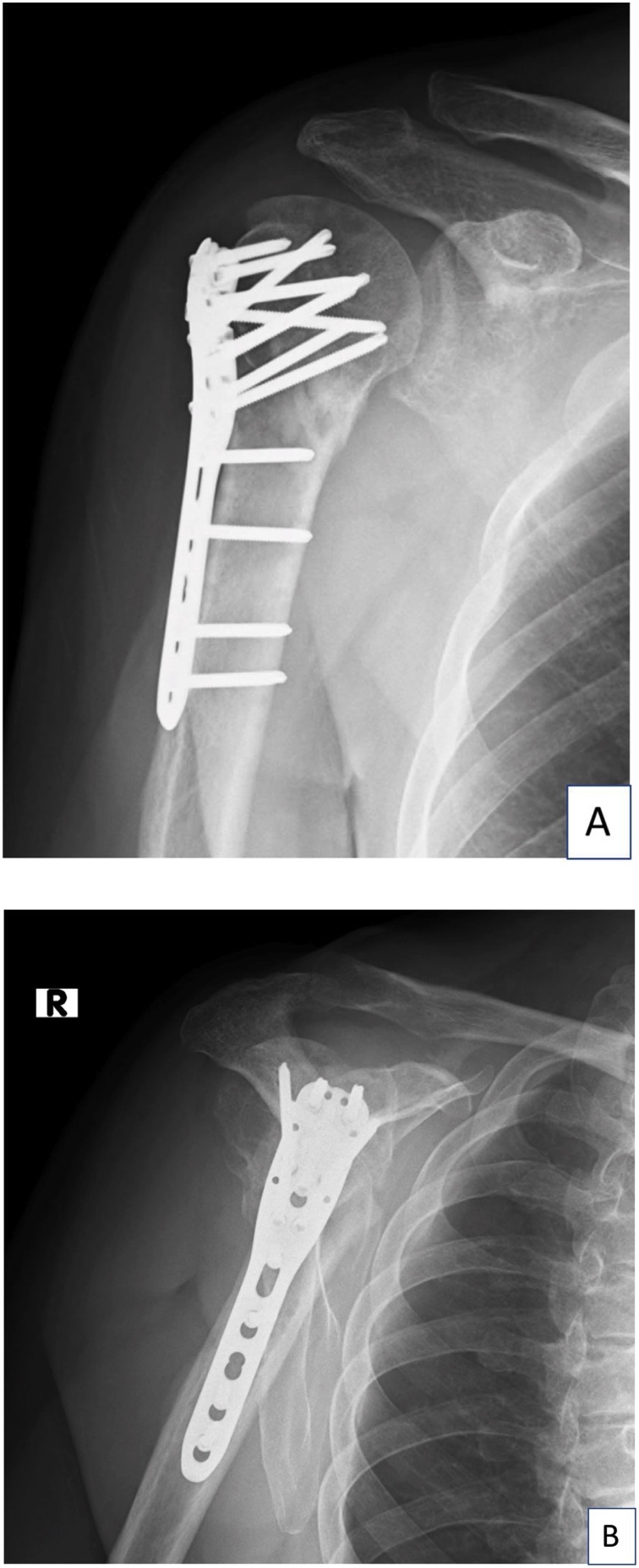
Radiographic imaging: (A) anteroposterior view of right shoulder, and (B) transcapular view of right shoulder at 6-month follow up.

A 40-year-old Thai male experienced right shoulder pain after a cycling accident. After the accident, he was brought immediately to the emergency room at our hospital. A primary survey according to the ATLS guidelines revealed no life-threatening injuries. His physical examination revealed a contusion over the right deltoid area (Fig. 1A, B). The maximal pain area was at the proximal humerus and he also complained of pain over the AC joint and at the mid-shaft area of the clavicle. The range of motion of his right shoulder was limited due to pain. The patient was sent for X-rays and a computer tomography (CT) scan in the supine position, both of which indicated only a proximal humerus fracture (Neer’s four parts) (Figs. 2A–C and 3A–C). Open reduction and internal fixation with a Philos plate (Synthes®) were performed with the patient in the supine position. During the operation, the AC joint injury was not noticed on the fluoroscope. One day after the surgery, the patient was sent for post-operative X-rays taken with the patient standing, which showed good alignment of fixation, but at this time the AC joint injury (Rockwood type III) was detected (Fig. 4A–C). We discussed the choices of treatment with the patient, who decided on conservative treatment with an arm sling. At his 6-month follow-up, the x-ray showed appropriate union of the right proximal humerus but the right acromioclavicular joint was separated (Fig. 5A–B).

## 3 Discussion

Proximal humerus fractures or AC joint injuries commonly occur after high-energy accidents. The most common mechanism of injury of these fractures is direct impact to the shoulder. In this case, the patient had a combined proximal humerus fracture and AC joint injury. Initially, we did not notice the AC joint injury because we were focused on the evident proximal humerus fracture and the images were done with the patient only in the supine position and showed only the proximal humerus fracture. After evaluation, open reduction and internal fixation were performed, and we missed the AC joint injury. We later realized the key reason we missed this injury was that there was no gravitational force during the image acquisition or our operation fluoroscopy which would reveal the AC joint displacement. This is a known phenomenon, as Pogorzelski et al. reported that AC joint injuries, especially the Rockwood types II and III, are often missed in a patient in the supine position because of no arm weight to affect the coracoclavicular distance [5]. Thus, in patients with a proximal humerus fracture, we recommend ensuring X-rays are taken or fluoroscopy performed with the patient in the upright, semi-upright or standing positions before surgery.

## 4 Conclusion

The combination of proximal humerus fracture and acromioclavicular (AC) joint injury is uncommon and has not been reported before. In the case of a patient with a proximal humerus fracture from a high-energy mechanism, the surgeon should acquire X-rays or fluoroscopic images with the patient in the upright or semi-upright position before surgery to avoid missing an AC joint injury, as delayed treatment can result in poor clinical outcomes.

## Funding

No funding was involved regarding this case report.

## Ethical approval

The present study was approved by the Prince of Songkla University Institutional Review Board, Faculty of Medicine, Songklanagarind Hospital, Prince of Songkla University (IRB number REC 62-411-11-1).

## Consent

Written informed consent was obtained from the patient for publication of this case report and accompanying images. A copy of the written consent is available for review by the Editor-in-Chief of this journal on request.

## Author contribution

Chaiwat Chuaychoosakoon —Preparation of case report, Literature review, Writing the paper.

Prapakorn Klabklay —Preparation of case report. Writing the paper.

## Registration of research studies

None.

## Guarantor

Chaiwat Chuayhoosakoon, MD.

## Provenance and peer review

Editorially reviewed, not externally peer-reviewed.

## Declaration of Competing Interest

No conflicts of interest.
